# Prevalence and factors associated with dental caries in patients attending an HIV care clinic in Uganda: a cross sectional study

**DOI:** 10.1186/s12903-019-0847-9

**Published:** 2019-07-19

**Authors:** Dunstan Kalanzi, Harriet Mayanja-Kizza, Damalie Nakanjako, Catherine Lutalo Mwesigwa, Ronald Ssenyonga, Bennett T. Amaechi

**Affiliations:** 1 Department of Dentistry School of Health Sciences, Makerere University College of Health Sciences, P.O.Box 7072, Kampala, Uganda; 2 Department of Medicine School of Medicine, Makerere University College of Health Sciences, Kampala, Uganda; 3 Department of Epidemiology and Biostatistics School of Public Health, Makerere University College of Health Sciences, Kampala, Uganda; 4 Department of Comprehensive Dentistry, University of Texas Health San Antonio School of Dentistry, San Antonio, TX USA

**Keywords:** Human immunodeficiency virus, Antiretroviral therapy, Dental caries

## Abstract

**Background:**

Chronic Human Immunodeficiency Virus (HIV) infection is associated with reduced saliva flow rate due to infiltration of HIV and proliferation of CD8 lymphocytes in salivary glands. It is unclear whether HIV infection and antiretroviral therapy (ART) increase caries risk. This study aimed to determine the prevalence and factors associated with dental caries in HIV infected adults attending the Mulago Immune Suppression Syndrome (ISS) clinic in Uganda.

**Methods:**

A cross-sectional study was conducted among HIV infected persons. Dental examinations were performed by two calibrated dentists using the WHO Radke’s caries classification criteria and reported using the decayed (D), missing (M), filled (F), teeth (DMFT) index. The prevalence and factors associated with dental caries was examined through linear regression analyses.

**Results:**

Overall, 748 participants (females = 491, 65.6%) with a mean age of 39 ± 9.4 years were included in the final analysis; of whom 83.7% had caries (DMFT> 0), with a significantly (*p* < 0.05) higher prevalence among females 86.6% than males 78.2%. The mean DMFT was 5.9 ± 5.5, with statistically significant differences based on gender (males 4.9 ± 4.8 and females 6.3 ± 5.9, p < 0.05) and duration on ART (< 2 years 4.8 ± 4.4, > 2 years but < 5 years 5.7 ± 5.5, > 5 years 6.6 ± 6.0 p < 0.05). The majority (67.2%) of participants reported brushing their teeth twice or more a day, and sugar intake was not associated with dental caries.

**Conclusion:**

Caries prevalence is high among HIV infected adults under care. Duration of ART was associated with increased risk and severity of caries. Therefore, we recommend integration of dental care in HIV treatment programs.

## Background

Dental caries is a major public health disease in all regions of the world [1]. People living with HIV (PLHIV) have a higher risk of developing dental caries compared to the general population [2, 3]. The major factors that have been suggested to increase the risk of dental caries in PLHIV are reduced saliva flow rate due to infiltration of HIV and proliferation of CD8 lymphocytes in salivary glands [4, 5] and ART use [6–8] that results in change in the normal microbial flora of the oral cavity. PLHIV are also at high risk for malnutrition [9], which may be exacerbated by symptoms of dental caries. Several studies in children [10–14] and the elderly [15, 16] show that dental caries is associated with poor nutritional outcomes. PLHIV are now living longer due to ART, which means that chronic health issues like dental caries are increasingly important to manage well in this patient population. However, there are paucity of data on the prevalence and factors associated with dental caries in HIV positive adults in sub-Saharan Africa (SSA). Therefore, the aim of the present study was to determine the prevalence of and factors associated with dental caries in this patient population, in order to inform the development of oral health care guidelines as a means of improving comprehensive adult HIV care.

## Methods

### Aim and design

This was a cross sectional study aimed at determining the prevalence and factors associated with dental caries among HIV infected adult patients.

### Study setting

The participants for this study were drawn from among HIV infected adults attending the Mulago ISS clinic (HIV care clinic) under the Makerere University Joint AIDS Program (MJAP). The Mulago ISS clinic has been in existence for about 15 years and is currently the largest HIV clinic in Kampala, Uganda’s capital city. The clinic provides comprehensive HIV services to over 16,000 adults, adolescents and children, 80% of whom are already on ART. This clinic is administered through the PEPFAR- supported Makerere University Joint AIDS Program and on average sees 300 patients on each working day.

### Sample size determination and power analysis

We used a two-group sample size for proportions to determine a sample size of 850 participants in order to detect a difference of 6% [17] between ART naïve (< 3 months on ART) and ART experienced (3+ months) with a power of 80 and 5% level of significance and an adjustment for a 10% non-response rate. The study was originally designed to test the hypothesis that caries experience among HIV infected ART naïve adults is different from that of HIV infected ART experienced adults. However, because of the change in HIV treatment care policy in Uganda to test and treat [18], we were not able to accrue sufficient numbers of HIV positive ART naïve participants for comparison purposes hence the use of the < 3 months on ART cut off as a proxy for ART naive. Further more, we conducted a power analysis during the analysis and found that with an 80% power and 5% level of significance, we could still detect a difference of as low as 8% with a sample size of 748.

### Study sample and participant recruitment

**Fig. 1:**
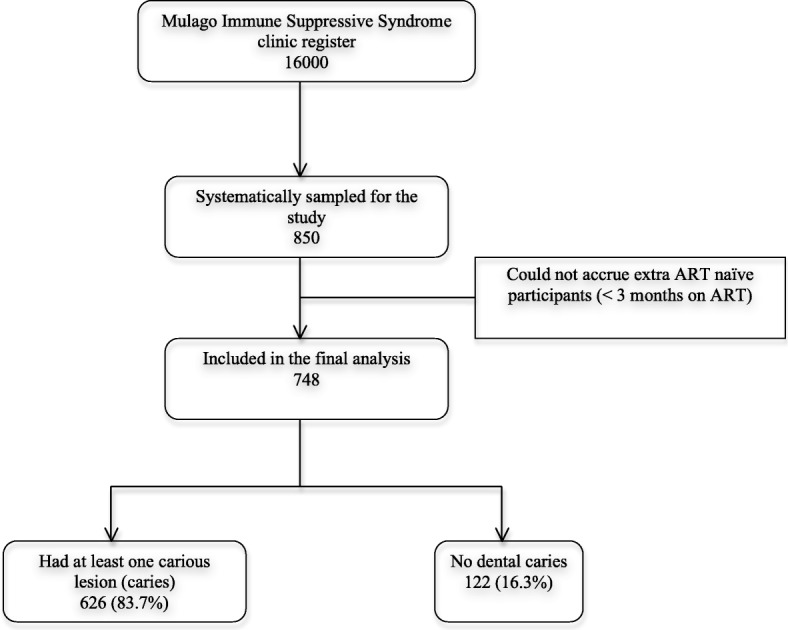
Study flow of participants

Participants aged 18 to 72 years were recruited from the Mulago ISS clinic. We used the systematic random sampling technique to enroll participants into the study. A total of 850 participants were targeted and this number was proportionately allocated to each clinic day over a period of three months as informed by the historical client clinic load. A sampling interval of 1–20 was determined from the need to enroll 15 participants from an average 300 participants that attended the clinic on any given day. A random number was selected from the sampling frame to determine the starting point of enrollment over the duration of the study recruitment (Fig. 1). HIV positive patients who agreed to participate in the study were brought to the Makerere University Dental school clinic by the study nurse, where written informed consents were obtained and study procedures performed. This process continued until the desired sample size was obtained.

### Data collection

Demographic and clinical data including age, gender, use of anti-retroviral medicine, xerostomia and smoking were captured using a modified World Health Organization (WHO) oral health questionnaire for adults [19] for each participant. Dental examinations were performed by two trained and calibrated dentists (DK and CLM) on dental chairs under suitable artificial light using a mirror and a probe, after wiping teeth using cotton roll to remove food debris. To ensure accurate and correct application of the research procedures through examiners’ practice, the two examiners were trained and calibrated by a benchmark examiner (CMR) on caries detection. The first 14 subjects that were recruited into the study were used for the calibration exercise. The agreement between the two examiners and calibrator, (interexaminer agreement) and the examiners’ individual repeated exercise (intraexaminer agreement) were evaluated using the unweighted kappa (κ) statistic. Caries was assessed using the WHO Radke’s caries classification criteria and reported using the decayed (D), missing (M), filled (F), teeth (DMFT) index as described by WHO [19]. A DMFT score of > 0 was considered as dental caries.

### Sugar intake

The frequency of sugar snacks consumption was assessed in terms of cakes/biscuits, bread/buns, jam/honey and sugar containing chewing gum intake. Sweet drink intake was assessed in terms of sugared tea /coffee and soft drinks. This is according to the WHO oral health questionnaire for adults. Sugar consumption was categorized as “less” representing seldom/never to once a week sugar intake and “more” representing several times a week to several times a day sugar intake.

### Data management and statistical methods

Data from completed questionnaires was keyed into pretested entry screens designed using EPIDATA software version 3.1. After validation, the data were then exported to STATA version 14 for analysis. Mean (SD) for age and proportions for categorical variables such as sex and residence were presented as descriptive statistics. Poisson regression models with robust standard errors and random effects estimator were fitted on the outcome of dental caries prevalence to generate incidence rate ratios interpreted as Prevalence Ratios (PR) for our study design and *P*-values in the assessment of association and statistical significance. Linear regression models were also fitted for the DMFT (severity) outcome via the generalized linearized model path. Inclusion of variables into the multivariable model for both analyses, was at a *p* value of < 0.2 or guidance from literature and confounding assessed at a 10% change in the prevalence ratios. Statistical significance was determined at two-sided *p*-values of less than 5%.

### Key study outcomes

Prevalence and severity as denoted by the DMFT index of dental caries (DMFT score of > 0 defined dental caries). Prevalence was assessed as a categorical variable whereas average DMFT was assessed as a continuous variable. Factors associated with dental caries in HIV infected adults were determined using linear regression.

## Results

### Calibration

The kappa statistic of over 0.73 across all rater comparisons denotes a strong agreement between the two raters, thus signifying a higher extent to which the data collected in this study are correct representations of the variables measured.

### Socio-demographics

Overall, 748 participants were included in the analysis. The mean age was 39 ± 9.4 years. Almost two thirds (491/748) of the participants were women and the majority (493/748) had attained some level of primary education. Over 50% (402/748) of the participants had been on ART for more than five years. The majority of participants reported good oral hygiene practices (506/746 (tooth brushing habit), 743/767 (toothbrush use), and 669/747 (toothpaste use)) and infrequent (seldom/never to once a week) consumption of sugar containing beverages and snacks (537/748). Only 3.5% (26/745) reported experiencing dry mouth symptoms (Table 1).

**Table 1:** Demographic data of randomly selected HIV infected adults attending an HIV care clinic in Uganda

Characteristic	*N* = 748 (%)
Age in years: mean	38.8 ± 9.4
Sex	
Male	257 (34.4)
Female	491 (65.6)
Residence	
Urban	204 (27.3)
Periurban	294 (39.3)
Rural	250 (33.4)
Education level	
No formal education	53 (7.1)
Primary	493 (65.9)
Secondary	149 (19.9)
Tertiary	53 (7.1)
Years on ART	
Less than 6 months	174 (23.3)
More than 6 months but less than 2 years	66 (8.8)
More than 2 years but less than 5 years	106 (14.2)
More than 5 years	402 (53.7)
Tooth brushing habit	
Seldom	13 (1.7)
Once a day	227 (30.4)
Twice or more	506 (67.8)
What is used to clean teeth^a^	
Toothbrush	743 (96.8)
Others (toothpicks, floss, charcoal)	24 (3.2)
Toothpaste use	
Toothpaste	669 (89.6)
Other	78 (10.4)
Dry mouth experience (self reported)	
No	719 (96.5)
Yes	26 (3.5)
Ever visited a dentist	
Yes	518 (69.3)
No	229 (30.7)
Sugar intake (Honey, Biscuits, Chew Gum, Soda)	
Less	537 (71.8)
More (Several times a week and more)	211 (28.2)

^a^ Multiple response variable

### Prevalence of dental caries

Among HIV infected individuals on ART, eight in every ten were found to have dental caries. Female gender, living in a rural setting, more than five years duration on ART and prior visit(s) to the dentist were associated with a higher caries burden and this difference was statistically significant (Table 2).

**Table 2:** Prevalence and Univariate analysis of risk factors for dental caries in HIV infected adults attending an HIV care clinic in Uganda

Characteristic	Had dental caries N (row %)		Unadjusted Prevalence Ratio (95% CI)	*P*-value
	Yes	No		
Overall	626 (83.7)	122 (16.3)		
Sex				
Male	201 (78.2)	56 (21.8)	1.00	
Female	425 (86.6)	66 (13.4)	1.26 (1.09,1.49)	< 0.05
Age in years				
18–24	34 (85.0)	6 (15)	1.00	
25–35	203 (83.9)	39 (16.1)	1.27 (0.89,1.81)	0.18
36–45	247 (81.0)	58 (19.0)	1.32 (0.93,1.87)	0.12
46–55	112 (86.8)	17 (13.2)	1.49 (1.03,2.16)	< 0.05
56+	30 (93.8)	2 (6.2)	1.90 (1.18,3.06)	< 0.05
Residence				
Urban	169 (82.8)	35 (17.2)	1.00	
Periurban	245 (83.3)	49 (16.7)	1.14 (0.94,1.37)	0.17
Rural	212 (84.8)	38 (15.2)	1.26 (1.04,1.52)	< 0.05
Education Level				
No formal education	44 (83.0)	9 (17.0)	1.00	
Primary	409 (83.0)	84 (17.0)	1.06 (0.79,1.42)	0.69
Secondary	126 (84.6)	23 (15.4)	0.97 (0.69,1.34)	0.85
Tertiary	47 (88.7)	7 (11.3)	1.12 (0.75,1.65)	0.59
Years on ART				
Less than 6 months	149 (85.6)	25 (14.4)	1.00	
More than 6 months but less than 2 years	56 (84.9)	10 (15.1)	0.94 (0.69,1.27)	0.69
More than 2 years but less than 5 years	86 (81.1)	20 (18.9)	1.17 (0.92,1.51)	0.20
More than 5 years	335 (83.3)	67 (16.7)	1.33 (1.11,1.60)	< 0.05
Tooth brushing habit				
Seldom	10 (76.9)	3 (23.1)	1.00	
Once a day	179 (78.8)	48 (22.2)	0.75 (0.42,1.32)	0.32
Twice or more	435 (86.0)	71 (14.0)	0.87 (0.49,1.53)	0.63
What is used to clean teeth				
Toothbrush	605 (83.7)	118 (16.3)	1.00	
Others (toothpicks, floss, charcoal)	20 (83.3)	4 (16.7)	1.25 (0.83,1.89)	0.29
Toothpaste use				
Toothpaste	557 (83.3)	112 (16.7)	1.00	
Other	68 (87.2)	10 (12.8)	1.15 (0.91,1.43)	0.23
Dry mouth experience (self-reported)				
No	600 (83.4)	119 (16.6)	1.00	
Yes	23 (86.5)	3 (11.5)	1.35 (0.91,2.01)	0.14
Ever visited a dentist				
Yes	509 (98.3)	9 (1.7)	1.00	
No	116 (50.7)	113 (49.3)	0.28 (0.78,1.08)	< 0.05
Sugar Intake (Honey, Biscuits, Soda)				
Less	442 (82.3)	95 (17.7)	1.00	
More (Several times a week and more)	184 (87.2)	27 (12.8)	0.92 (0.78,1.08)	0.30

As shown in Table 3, female gender was independently associated with a modest (21%) increase risk of dental caries. The lack of a prior visit to a dentist was independently associated with a 28% lower risk of dental caries.

**Table 3:** Multivariate analysis of risk factors for dental caries in HIV infected adults attending an HIV care clinic in Uganda

Characteristic	Univariate Model PR	Final multivariate model PR (95% CI)	*P*-value
Sex	1.28	1.21 (1.05,1.40)	< 0.05
Age in years	1.12	1.02 (0.95,1.10)	0.62
Residence	1.12	1.07 (0.98,1.16)	0.13
Education Level	0.99	0.94 (0.86,1.03)	0.21
Years on ART	1.11	1.04 (0.98,1.10)	0.18
Tooth brushing habit	1.10	1.00 (0.88,1.14)	0.96
Toothpaste use	1.16	1.08 (0.87,1.33)	0.48
Ever visited a dentist	0.28	0.28 (0.24,0.33)	< 0.05
Sugar Intake (Honey, Biscuits, Soda)	0.92	1.02 (0.88,1.18)	0.79

Modified Poisson regression model used for multivariate analysis

### Severity of dental caries

The severity of dental caries as denoted by DMFT was on average 5.9 ± 5.5. The average DMFT was higher in females (6.3) when compared to males (4.9) and this difference was statistically significant (*p* < 0.05). The average DMFT was significantly increased with age and duration on ART. Interestingly, the average DMFT was significantly lower among persons that had not been to the dentist (Table 4).

**Table 4:** Univariate analysis of risk factors for severity of dental caries in HIV infected adults attending an HIV care clinic in Uganda

Characteristic	Mean DMFT (SD)	Coefficients (95% CI)	*P*-value
Overall	5.9 (5.5)		
Sex			
Male	4.9 (4.8)	1.00	
Female	6.3 (5.9)	1.35 (0.52,2.19)	< 0.05
Age in years			
18–24	4.3 (4.6)	1.00	
25–35	5.6 (5.3)	1.26 (−0.58,3.11)	0.18
36–45	5.8 (5.3)	1.48 (−0.34,3.29)	0.11
46–55	6.4 (5.7)	2.1 (0.15,4.07)	0.04
56+	8.5 (8.6)	4.1 (1.58,6.71)	< 0.05
Residence			
Urban	5.2 (5.3)	1.00	
Periurban	5.9 (5.4)	0.69 (−0.29,1.69)	0.17
Rural	6.5 (5.9)	1.31 (0.29,2.34)	0.01
Education Level			
No formal education	5.7 (5.7)	1.00	
Primary	5.9 (5.7)	0.31 (−1.26,1.88)	0.70
Secondary	5.5 (5.2)	−0.17(−1.92,1.56)	0.84
Tertiary	6.3 (5.1)	0.68 (− 1.40,2.79)	0.53
Years on ART			
Less than 6 months	4.9 (4.6)	1.00	
More than 6 months but less than 2 years	4.5 (3.9)	−0.41(−1.97,1.12)	0.60
More than 2 years but less than 5 years	5.7 (5.5)	0.83 (− 0.49,2.16)	0.22
More than 5 years	6.6 (6.0)	1.64 (0.66,2.62)	< 0.05
Tooth brushing habit			
Seldom brush	7.2 (8.6)	1.00	
Once a day	5.2 (5.3)	−1.94 (−5.04,1.16)	0.22
Twice or more	6.1 (5.5)	−1.01 (−4.06,2.04)	0.52
What is used to clean teeth			
Toothbrush	5.8 (5.5)	1.00	
Others (toothpicks, floss, charcoal)	7.3 (7.2)	1.50 (−0.75,3.76)	0.19
Toothpaste use			
Toothpaste	5.8 (5.6)	1.00	
Other	6.7 (5.5)	0.87 (−0.43,2.17)	0.19
Dry mouth experience (Self-reported)			
No	5.8 (5.5)	1.00	
Yes	7.8 (7.8)	2.00 (−0.17,4.18)	0.07
Ever visited a dentist			
Yes	7.6 (5.5)	1.00	
No	2.1 (3.4)	−5.47(−6.24, 4.71)	< 0.05
Sugar Intake (Honey, Biscuits, Soda)			
Less	6.0 (5.8)	1.00	
More (Several times a week and more)	5.5 (4.8)	−0.48 (−1.36,0.41)	0.29

Severity of dental caries is denoted by the DMFT (Decayed, Missing, Filled Teeth) index

As shown in Table 5, female gender, > 5 years of ART, and prior visit to the dentist were independently associated with an increased risk of more severe dental caries.

**Table 5:** Multivariate analysis of risk factors for severity dental caries in HIV infected adults attending an HIV care clinic in Uganda

Characteristic	Univariate Model coefficient	Final multivariate model coefficient (95% CI)	*P*-value
Sex	1.35	0.99 (0.20,1.79)	< 0.05
Age in years	0.68	0.19 (−0.24,0.63)	0.37
Residence	0.65	0.29 (−0.19,0.76)	0.24
Education Level	0.01	−0.14(− 0.67,0.39)	0.59
Years on ART	0.06	0.34 (0.03,0.65)	< 0.05
Tooth brushing habit	0.61	0.22 (−0.48,0.93)	0.53
Toothpaste use	0.87	0.51 (−0.67,1.69)	0.39
Ever visited a dentist	−5.50	−5.29(−6.01,4.51)	< 0.05
Sugar Intake (Honey, Biscuits, Soda)	−0.40	− 0.002(− 0.83,0.82)	0.99

## Discussion

The present study investigated the prevalence, severity and risk factors of dental caries among HIV infected patients attending the Mulago ISS clinic in Uganda. The prevalence and severity of dental caries among HIV infected patients was high. More severe dental caries in HIV patients was associated with the female gender, duration on ART, self-reported experience of dry mouth and prior visit to the dentist. These findings are not surprising considering that it is well known that dental caries is a major public health disease in all regions of the world and one of the major causes of tooth loss [1]. In 2010, 2.4 billion people across the globe were affected by dental caries [20] and the greatest burden normally being among the disadvantaged and socially marginalized populations [19]. Therefore, the proportion (84%) of dental caries in this study population supports these observations.

The finding of the present study is in agreement with previous studies that reported PLHIV to have a higher risk of developing dental caries [8, 21, 22] compared to the general population. However, studies on dental caries rate among HIV patients on ART are scarce while the only available one did not show any specific trend [23]. Although our study did not compare dental caries among HIV positive versus negative patients, the prevalence and severity of caries in PLHIV was higher in reference to a survey of the general Ugandan population. The prevalence and severity in the general adult Ugandan population was reported to be 66.7% and 4.71 respectively [24].

Salivary gland hypofunction and medications are among the factors suggested to increase dental caries in HIV [8, 21, 25]. This present study noted that a higher proportion of those participants experiencing dry mouth had significantly higher mean DMFT than those who did not report the same experience. This is in agreement with a long-established fact that low saliva flow rate as seen in dry mouth conditions (xerostomia) places an individual at a higher risk of developing dental caries [26, 27]. On the same footing, it is not surprising that in the present study, participants on ART for more than five years have a higher DMFT score than other groups, considering the well-established knowledge that 80% of prescribed drugs cause xerostomia [26, 27].

The increased risk of dental caries with age is not surprising since the DMFT index is a measure of caries experience, which naturally increases with age. However, the higher risk among participants that had a prior visit to the dentist might be due to the fact that the population under study has poor oral health seeking behaviours, which has been reported in other African populations [28]. In such settings, dental care is usually emergency care in response to pain. Therefore, without pain individuals have no reason to seek dental care or might be faced by other access challenges.

PLHIV are also at a higher risk for malnutrition [9], which may be exacerbated by dental caries. Studies suggest that caries of the primary and permanent dentitions is associated with early childhood malnutrition [12, 14] and increased likelihood of malnutrition [15] in the elderly respectively. PLHIV are now living longer due to ART, which means that chronic health issues like dental caries and oral health are increasingly important to manage well in HIV patients to prevent complications such as malnutrition.

Our study limitations were as follows:

- Considering that DMFT index does not record teeth with incipient caries as decayed, it is possible that many-decayed teeth with early caries were unaccounted for.
- The lack of an HIV negative comparison group.
- Survival bias might have affected the association between severity of caries and duration on ART and the proxy used for xerostomia was incomplete for the majority of participants as saliva flow rates were only done for 168 participants involved in a sub-study.
  Therefore, we suggest that future studies should employ a more robust caries classification system such as the International Caries Detection and Assessment System (ICDAS II) [29] for caries assessment. We also recommend longitudinal studies involving both HIV positive and negative individuals.

## Conclusions

The prevalence of dental caries was high (83.7%) among HIV infected adults under care and longer duration of ART was associated with increased risk of caries. Therefore, we recommend integration of dental care in HIV treatment programs to further understand the HIV-associated biological and behavioural risk of caries.

## Data Availability

The datasets used and/or analyzed during the current study are available from the corresponding author on reasonable request.

## Abbreviations

ART: Antiretroviral Therapy
DMFT: Decayed, Missing, Filled Teeth
HIV: Human Immunodeficiency Virus
ICDAS II: International Caries Detection and Assessment System
ISS: Immune Suppression Syndrome
PLHIV: People Living with HIV
SSA: Sub-Saharan Africa
WHO: World Health Organization

## References

[1] Petersen PE, Bourgeois D, Ogawa H, Estupinan-Day S, Ndiaye C (2005). The global burden of oral diseases and risks to oral health. Bull World Health Organ.

[2] Goldberg BE, Mongodin EF, Jones CE, Chung M, Fraser CM, Tate A, Zeichner SL (2015). The Oral bacterial communities of children with well-controlled HIV infection and without HIV infection. PLoS One.

[3] Nouaman MN, Meless DG, Coffie PA, Arrive E, Tchounga BK, Ekouevi DK, Anoma C, Eholie SP, Dabis F, Jaquet A (2015). Oral health and HIV infection among female sex workers in Abidjan, cote d'Ivoire. BMC Oral Health.

[4] Itescu S, Brancato LJ, Buxbaum J, Gregersen PK, Rizk CC, Croxson TS, Solomon GE, Winchester R (1990). A diffuse infiltrative CD8 lymphocytosis syndrome in human immunodeficiency virus (HIV) infection: a host immune response associated with HLA-DR5. Ann Intern Med.

[5] Mandel L, Surattanont F (2002). Regression of HIV parotid swellings after antiviral therapy: case reports with computed tomographic scan evidence. Oral Surg Oral Med Oral Pathol Oral Radiol Endod.

[6] Navazesh M, Mulligan R, Barron Y, Redford M, Greenspan D, Alves M, Phelan J, Women's Interagency HIVS (2003). A 4-year longitudinal evaluation of xerostomia and salivary gland hypofunction in the Women's Interagency HIV study participants. Oral Surg Oral Med Oral Pathol Oral Radiol Endod.

[7] Nittayananta W, Chanowanna N, Jealae S, Nauntofte B, Stoltze K (2010). Hyposalivation, xerostomia and oral health status of HIV-infected subjects in Thailand before HAART era. J Oral Pathol Med.

[8] Nittayananta W, Talungchit S, Jaruratanasirikul S, Silpapojakul K, Chayakul P, Nilmanat A, Pruphetkaew N (2010). Effects of long-term use of HAART on oral health status of HIV-infected subjects. J Oral Pathol Med.

[9] Duggal S, Chugh TD, Duggal AK (2012). HIV and malnutrition: effects on immune system. Clin Dev Immunol.

[10] Janakiram C, Antony B, Joseph J (2018). Association of Undernutrition and Early Childhood Dental Caries. Indian Pediatr.

[11] Ribeiro CC, da Silva MC, Machado CM, Ribeiro MR, Thomaz EB (2014). Is the severity of caries associated with malnutrition in preschool children?. Cien Saude Colet.

[12] So M, Ellenikiotis YA, Husby HM, Paz CL, Seymour B, Sokal-Gutierrez K (2017). Early childhood dental caries, mouth pain, and malnutrition in the Ecuadorian Amazon region. Int J Environ Res Public Health.

[13] Aluckal E, Anzil K, Baby M, George EK, Lakshmanan S, Chikkanna S (2016). Association between body mass index and dental caries among Anganwadi children of Belgaum City, India. J Contemp Dent Pract.

[14] Psoter WJ, Reid BC, Katz RV (2005). Malnutrition and dental caries: a review of the literature. Caries Res.

[15] Wu LL, Cheung KY, Lam PYP, Gao XL (2018). Oral health indicators for risk of malnutrition in elders. J Nutr Health Aging.

[16] Baumgartner W, Schimmel M, Muller F (2015). Oral health and dental care of elderly adults dependent on care. Swiss Dent J.

[17] Rwenyonyi CM, Kutesa A, Muwazi L, Okullo I, Kasangaki A, Kekitinwa A (2011). Oral manifestations in HIV/AIDS-infected children. Eur J Dent.

[18] UNAIDS: http://www.unaids.org/en/resources/presscentre/featurestories/2018/april/test-and-treat-showing-results-in-uganda-and-zambia. 2018. Accessed 15 July 2019.

[19] Petersen PE, Baez RJ, World Health Organization. Oral health surveys: basic methods. 5th Edition. World Health Organization; 2013.

[20] Frencken JE, Sharma P, Stenhouse L, Green D, Laverty D, Dietrich T (2017). Global epidemiology of dental caries and severe periodontitis - a comprehensive review. J Clin Periodontol.

[21] Cavasin Filho JC, Giovani EM (2009). Xerostomy, dental caries and periodontal disease in HIV+ patients. Braz J Infect Dis.

[22] Aleixo Rodrigo Queiroz, Scherma Alexandre Prado, Guimarães Gustav, Cortelli José Roberto, Cortelli Sheila Cavalca (2010). DMFT index and oral mucosal lesions associated with HIV infection: cross-sectional study in Porto Velho, Amazonian region - Brazil. Brazilian Journal of Infectious Diseases.

[23] Rezaei-Soufi L, Davoodi P, Abdolsamadi HR, Jazaeri M, Malekzadeh H (2014). Dental caries prevalence in human immunodeficiency virus infected patients receiving highly active anti-retroviral therapy in Kermanshah, Iran. Cell J.

[24] Kutesa A, Kasangaki A, Nkamba M, Muwazi L, Okullo I, Rwenyonyi CM (2015). Prevalence and factors associated with dental caries among children and adults in selected districts in Uganda. Afr Health Sci.

[25] Navazesh M, Mulligan R, Karim R, Mack WJ, Ram S, Seirawan H, Greenspan J, Greenspan D, Phelan J, Alves M (2009). Effect of HAART on salivary gland function in the Women's Interagency HIV study (WIHS). Oral Dis.

[26] Saini T, Edwards PC, Kimmes NS, Carroll LR, Shaner JW, Dowd FJ (2005). Etiology of xerostomia and dental caries among methamphetamine abusers. Oral Health Prev Dent.

[27] Murray Thomson W (2014). Epidemiology of oral health conditions in older people. Gerodontology.

[28] Varenne B, Petersen PE, Fournet F, Msellati P, Gary J, Ouattara S, Harang M, Salem G (2006). Illness-related behaviour and utilization of oral health services among adult city-dwellers in Burkina Faso: evidence from a household survey. BMC Health Serv Res.

[29] Dikmen B (2015). Icdas II criteria (international caries detection and assessment system). J Istanb Univ Fac Dent.

